# The Impact of Age on Propofol Requirement for Inducing Loss of Consciousness in Elderly Surgical Patients

**DOI:** 10.3389/fphar.2022.739552

**Published:** 2022-03-28

**Authors:** Hua Yang, Hui-Min Deng, Hai-Yan Chen, Shu-Heng Tang, Fang Deng, Yu-Gang Lu, Jin-Chao Song

**Affiliations:** ^1^ Department of Anesthesiology, Shidong Hospital Affiliated to University of Shanghai for Science and Technology, Shanghai, China; ^2^ Department of Anesthesiology, Shanghai Pulmonary Hospital, School of Medicine, Tongji University, Shanghai, China

**Keywords:** elderly, anesthetics, propofol, intravenous anaesthesia, loss of consciousness

## Abstract

It is generally accepted that geriatric patients are more sensitive to propofol than adults; thus, a dose-adjusted propofol is recommended for these patients during the induction of anesthesia. However, for patients aged 75 years and over, established guidelines for propofol induction doses do not provide dose references. To this end, we observed 80 surgical patients (female 39, male 41, American Society of Anesthesiologists physical status score I ∼ II) to access the appropriate dose of propofol for inducing loss of consciousness (LOC). Accordingly, patients were subdivided into group A (20 patients, 45–64 years), group B (20 patients, 65–74 years), group C (20 patients, 75–84 years), and group D (20 patients, ≥ 85 years). All patients received propofol (at a rate of 0.3 mg/kg/min) alone for inducing LOC, which was defined by loss of both eyelash reflex and verbal response. Compared with group A, the propofol requirement for LOC in Group B, C and D decreased by 14.8, 25.2 and 38.5%, respectively. Bivariate linear correlation analysis showed that propofol requirement was negatively correlated with age. After adjusting for potential confounders, age was still an independent factor affecting propofol requirement. In conclusion, the propofol requirement for inducing LOC decreased significantly in elderly patients. We demonstrated that age was an independent factor impacting propofol requirement for LOC during the induction of general anesthesia, implying that the propofol dose for anesthesia induction should be further reduced in elderly surgical patients, especially those aged 75 years and over.

## Introduction

Advances in surgical techniques and improvement of perioperative management have led to a larger proportion of elderly patients presenting to undergo surgical procedures (Aurini and White, 2014). Previous studies have shown that more than half of all surgical procedures were performed on patients over the age of 65 (Yang et al., 2011). With the deepening of global aging, this proportion is expected to further increase in the coming decades. For these patients, anesthesiologists often need to adjust the anesthetic regimen, including medication selection, dosage optimization and so on, to adapt to the elderly physiological changes. However, only limited empirical data on guiding the appropriate dosing of anesthetic induction agents for elderly patients can be referred by the anesthesiologists (Phillips et al., 2015).

Propofol, as an intravenous hypnotic agent, has been widely used for anesthetic induction and maintenance in surgical patients. It can provide quick and smooth anesthesia induction. However, a common side-effect of propofol-based induction is dose-dependent hemodynamic instability (Sahinovic et al., 2018), such as hypotension and bradycardia, especially in elderly patients. Compared with the middle-aged adult, the principles of geriatric physiology are not merely a linear extension (Yang et al., 2011). These elderly patients represent a unique clinical group, in addition, they typically suffer from a number of chronic diseases. Although some studies have recommended that propofol-based induction should be avoided in these elderly individuals (Reich et al., 2005; Phillips et al., 2015), many anesthesiologists still choose to use propofol in clinical anesthesia rather than other drugs which have little effect on hemodynamics.

Based on the fact that elderly patients have increased sensitivity to propofol, anesthesiologists are recommended to reduce the dose of propofol used for induction in patients aged over 65 years from 2 to 2.5 mg/kg to 1–1.5 mg/kg (McEvoy et al., 2008). However, for patients over 75 or even 85 years old, it is unclear whether this recommendation is still applicable and what dose of propofol is appropriate for such patients. To this end, we designed the current study to access the appropriate dose of propofol for these patients, and to analyze the role of age on propofol requirement in the process of loss of consciousness (LOC) induced by propofol.

## Patients and Methods

### Participants

We conducted a cross-sectional study following the Declaration of Helsinki from April to August 2020. The study was approved by the Institutional Research Ethics Committee of Shidong Hospital. Patients aged 45 years and over scheduled for general surgery or orthopedic surgery under general anesthesia were considered eligible. Patients were excluded if they: = 1 \* GB3 ① American Society of Anesthesiologists (ASA) physical status score ≥3; = 2 \* GB3 ② allergic to propofol; = 3 \* GB3 ③ body mass index (BMI) ≤ 20 or ≥30 kg/m^2^; = 4 \* GB3 ④ taking hypnotics, opioid analgesic or antianxiety agents; = 5 \* GB3 ⑤ known or suspected heart failure (ejection fraction <40%), severe respiratory disease, renal or metabolic diseases; = 6 \* GB3 ⑥ could not complete the informed consent procedure independently. Written informed consent was obtained from all patients.

### Study Protocol

A total of 80 patients who met the inclusion and the exclusion criteria were divided into four groups, Group A (20 patients, 45–64 years), Group B (20 patients, 65–74 years), Group C (20 patients, 75–84 years), and Group D (20 patients, ≥ 85 years), according to age. Noninvasive blood pressure, heart rate, electrocardiogram, pulse oxygen saturation and end-tidal carbon dioxide were monitored continuously throughout the operation. After 5 min of preoxygenation, propofol was pumped at a rate of 0.3 mg/kg/min until the LOC occurred. The LOC was defined by loss of both eyelash reflex and verbal response. The assessment of the loss of eyelash reflex and verbal response was carried out every 10 s after propofol pumping for 1.5 min. An anesthesiologist assistant, who was blinded to the grouping, performed the above reflex assessment and finally determined the end point of titration. Meanwhile, the dose of propofol (Propofol requirement) and the time of reflection disappear (T reflection disappear) for each patient were recorded. After induction of propofol, 0.4–0.6 ug/kg of sufentanil and 0.2 mg/kg of cisatracurium were administered, and endotracheal intubation was performed 3 min later.

Perioperative variables included in the analysis were sex, BMI, albumin (ALB), total bilirubin (TBIL), alanine aminotransferase (ALT), aspartate aminotransferase (AST), serum creatinine (Scr), blood urea nitrogen (BUN), glomerular filtration rate (GFR), ejection fraction (EF) and pulse oxygen saturation (SpO2).

Hemodynamic parameters including mean arterial pressure (MAP) and heart rate (HR) at five different time points (T0, before propofol administration; T1, LOC; T2, 3 min after the administration of fentanyl and cisatracurium; T3, 1 min after intubation; T4, 5 min after intubation; T5, 10 min after intubation) were recorded. Hypotension was defined as a MAP <65 mmHg, hypertension was defined as a MAP >90 mmHg, bradycardia was defined as a HR < 45 bpm persisting more than 30 s and was treated with IV atropine 0.5 mg.

### Statistical Analysis

Baseline characteristics of patients were described as mean (standard deviation, SD) for continuous variables, frequency (percentage) for categorical variables, or median (interquartile range, IQR) for continuous variables with skewed distribution. One-way analysis of variance, Kruskal–Wallis test and χ^2^ test were used to analyze the demographic data and hemodynamic changes in each group, as appropriate. We used the Mantel-Haenszel χ2 test to explore the trend relationship between categorical variables and age groups, and linear regression between continuous variables and age groups. Pearson correlation coefficients were calculated to assess correlations between propofol requirement and different parameters.

To evaluate the independent association of age with propofol requirement, multiple linear regression models were constructed. Three models were fitted, Model I: unadjusted; Model II: adjusted for gender and BMI; Model III: further adjusted for, ALT, ALB, and GFR. The selection of variables in the model was based on univariate analysis results at *p-*value < 0.1 and clinical expertise to assess whether variables within the model affected drug metabolism *in vivo*. Furthermore, considering the strong professional relevance of several indicators of liver or kidney function, we selected those that met the clinical value to enter the model. The collinearity diagnostic was used to determine whether the variables in the model were highly interrelated, as determined by the variance inflation factor and tolerance. We also use smooth curve fitting to examine whether the relationship between age and propofol requirement was linear while adjusting for potential confounders above. All statistical analyses were performed using SPSS, version 20.0 (SPSS Inc. Chicago, IL, United States) and R, version 3.6.3 (R Project for Statistical Computing). A two-side *p*-value < 0.05 was considered statistically significant.

### Sensitivity Analysis

Given the limitations of the study sample size, we observed the robustness of the findings by including different indicators of kidney and liver function into the models separately, and assessed the model fit by *R* ^2^ and adjusted *R* ^2^. Model I adjusted for gender, BMI, ALT, and GFR; model II adjusted for gender, BMI, ALT, GFR, ALB, and TBIL; model III adjusted for gender, BMI, ALT, GFR, ALB, TBIL, and AST.

## Results

The baseline characteristics of patients are presented in Table 1. Of the 80 patients (mean age = 74 ± 12 years, range 45–93 years), 39 (48.75%) were female. No differences in BMI, ALT, AST, BUN, EF and SpO2 between age groups. However, GFR was significantly associated with age (*p* = 0.014) and the mean difference (95%CI) in age between groups A and B was 29.59 (4.60–54.58). Furthermore, GRF and albumin decreased linearly with age, whereas Scr and bilirubin were reversed (all *p*-trends < 0.05).

**TABLE 1:** Baseline characteristics of patients in different age groups.

	Group A (n = 20)	Group B (n = 20)	Group C (n = 20)	Group D (n = 20)	*p* Value	*p* Trend
Male, No. (%)	9 (45)	10 (50)	11 (55)	11 (55)	0.908	0.487
BMI, mean (SD), kg/m^2^	24.05 (0.61)	24.40 (0.50)	24.05 (0.61)	24.40 (0.50)	0.055	0.224
Liver function						
Albumin, mean (SD), g/L	42.15 (7.08)	41.13 (5.04)	39.32 (4.00)	37.76 (7.74)	0.121	0.016
Bilirubin, median (IQR), μmol/L	11.50 (9.70–15.78)	12.90 (10.25–19.83)	14.85 (12.13–22.45)	15.30 (11.60–23.18)	0.123	0.034
ALT, median (IQR), U/L	18.50 (11.25–26.50)	18.00 (12.00–22.75)	12.50 (11.00–18.25)	14.50 (10.25–24.00)	0.358	0.583
AST, median (IQR), U/L	22.00 (18.00–25.00)	23.00 (16.00–32.00)	21.00 (17.00–28.50)	22.00 (17.25–28.25)	0.958	0.168
Kidney function						
Scr, median (IQR), mg/dl	0.67 (0.55–0.86)	0.80 (0.68–0.92)	0.80 (0.65–1.01)	0.78 (0.62–1.05)	0.126	0.033
BUN, median (IQR), mg/dl	14.57 (11.90–16.95)	15.55 (12.74–19.54)	13.98 (10.99–17.44)	15.69 (11.25–20.02)	0.746	0.761
GFR, mean (SD), mL/min/1.73m^2^	136.83 (29.07)	115.41 (26.87)	107.24 (28.67)	111.98 (35.09)	0.014	0.008
EF, mean (SD) %	63.65 (2.37)	62.35 (1.90)	64.05 (3.32)	63.10 (3.16)	0.239	0.986
SpO2, mean (SD) %	97.25 (0.91)	96.60 (0.99)	96.70 (0.98)	96.85 (0.88)	0.146	0.252

BMI, body mass index; ALT, alanine aminotransferase; AST, aspartate aminotransferase; Scr, serum creatinine, BUN, blood urea nitrogen; GFR, glomerular filtration rate; EF, ejection fraction; SpO2, pulse oxygen saturation.

*p* value in one-way analysis of variance, Kruskal-Wallis test, or χ2 test; *p* trend in linear regression or Mantel-Haenszel χ2 test.

**FIGURE 1:**
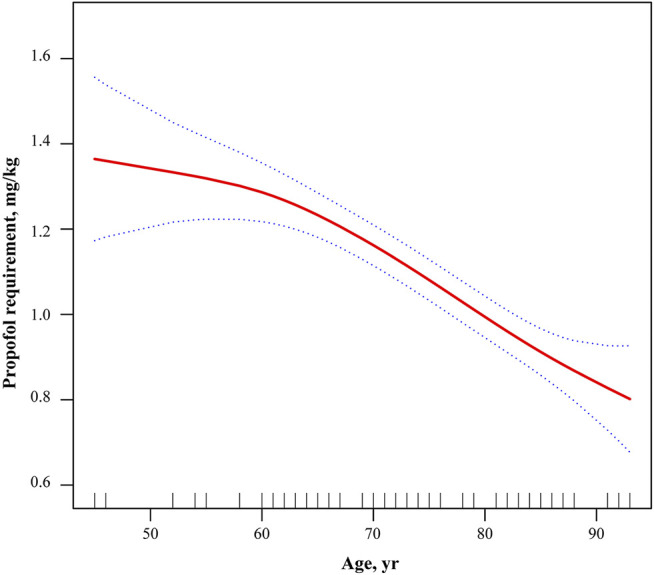
Adjusted dose-response relationship between propofol requirement and age. Adjusted for gender, BMI, ALB, ALT, and GFR. BMI, body mass index; ALB, albumin; ALT, alanine aminotransferase; AST, aspartate aminotransferase; GFR, glomerular filtration rate.

Differences in anesthesia effects between ages were shown in Table 2. Propofol requirement and T reflection disappear differed significantly between ages (*p* < 0.001), and all had significant linear decreases with age (P-trend < 0.001). Compared to group A, group B had a mean reduction in propofol requirement of 0.20 mg/kg (95%CI = 0.07–0.33) and a mean reduction in T reflection disappear of 32.50 s (95%CI = 7.28–57.72). Significant differences were consistent across all neighboring groups (all *p* < 0.05). Additionally, to further determine whether the relation between age and propofol requirement was linear, the estimated dose-response curve was fitted. There was a continuous linear decreasing trend and statistical significance between propofol requirement and age after adjusting for gender, BMI, ALB, ALT, and GFR (Figure 1).

**TABLE 2:** Comparison of anesthetic effects in different age groups.

	Group A (*n* = 20)	Group B (*n* = 20)	Group C (*n* = 20)	Group D (*n* = 20)	*p* Value	*p* Trend
Propofol requirement, mean (SD), mg/kg	1.35 (0.20)	1.15 (0.20)^a^	1.01 (0.21)^b^	0.83 (0.17)^c^ ^,^ ^d^ ^,^ ^e^	< 0.001	< 0.001
T reflection disappear, mean (SD), s	262.50 (36.11)	230.00 (42.43)^a^	200.50 (43.34)^b^	165.00 (32.36)^c^ ^,^ ^d^ ^,^ ^e^	< 0.001	< 0.001

a Significant difference between Group A and B.

b Significant difference between Group A and C.

c Significant difference between Group A and D.

d Significant difference between Group B and D.

e Significant difference between Group C and D.

Bivariate linear correlation analysis showed that propofol requirement was significantly and positively correlated with albumin (r = 0.312; 95% CI = 0.099–0.497; *p* = 0.005) and GRF (r = 0.286; 95%CI = 0.070–0.476; *p* = 0.010), and negatively correlated with age, such that significant decline in propofol requirement with increasing age (r = -0.689; 95%CI = -0.789 ∼ -0.553; *p* < 0.001). Other kidney and liver parameters were not significantly correlated with propofol requirements (Table 3).

**TABLE 3:** Correlation coefficients between age and propofol requirement.

	Propofol Requirement, Mg/Kg		
	R	95% CI	*p* Value
Age, yr	−0.689	−0.789–0.553	< 0.001
BMI, kg/m^2^	−0.214	−0.414–0.006	0.057
Albumin, g/L	0.312	0.099–0.497	0.005
Ln (Bilirubin), μmol/L	−0.169	−0.375–0.052	0.133
Ln (ALT), U/L	0.018	−0.203–0.237	0.875
Ln (AST), U/L	−0.145	−0.353–0.078	0.201
Ln (Scr), mg/dl	−0.173	−0.379–0.048	0.124
Ln (BUN), mg/dl	0.037	−0.184–0.255	0.744
GFR, mL/min/1.73m^2^	0.286	0.070–0.476	0.010
EF, %	0.095	−0.127–0.308	0.401

BMI, body mass index; ALT, alanine aminotransferase; AST, aspartate aminotransferase; Scr, serum creatinine, BUN, blood urea nitrogen; GFR, glomerular filtration rate; EF, ejection fraction.

Patient age was an independent and significant factor in propofol requirement. When propofol requirement entered models as a continuous variable, advanced age was associated with lower propofol requirements. In the fully adjusted model, none of the variables included in the model were strongly interrelated by collinearity diagnostic. per 1-SD increase in age was associated with a decrease in propofol requirement of approximately 0.171 (*β* = −0.167; 95%CI = −0.218 ∼ −0.116; *p* < 0.001). In the unadjusted model, a high level of age (Group D) was strongly associated with a lower propofol requirement (*β* = −0.525; 95%CI = −0.648 ∼ −0.403; *p* < 0.001). Furthermore, P-trend was calculated using age groups as ordinal variables, and the results showed a linear trend between age and propofol requirement (P-trend < 0.001). The association yielded relatively consistent results after adjusting for gender and BMI [β (95%CI): Group B: 0.185 (−0.310 ∼ −0.060), *p* = 0.004; Group C: 0.348 (−0.470 ∼ −0.226), *p* < 0.001; Group D: 0.512 (−0.637 ∼ −0.387), *p* < 0.001] (Model II). After further adjustment for, ALT, ALB, and GFR, the associations were slightly weakened but still statistically significant, with β values of −0.158 (95%CI = −0.286 ∼ −0.029; *p* = 0.017), −0.316 (95%CI = −0.449 ∼ −0.182; *p* < 0.001) and −0.459 (95%CI = −0.595 ∼ -0.324; *p* < 0.001) for groups B, C, and D, respectively (Model III) (Table 4).

**TABLE 4:** Effects of age on propofol requirement.

Age, yr	Model I		Model II		Model III	
	β (95%CI)	*p* Value	β (95%CI)	*p* Value	β (95%CI)	*p* Value
Per 1 SD	−0.187 (−0.232 ∼ −0.143)	< 0.001	−0.185 (−0.230 ∼ −0.140)	< 0.001	−0.167 (−0.218 ∼ −0.116)	< 0.001
Group A	0.00 [References]	—	0.00 [References]	—	0.00 [References]	—
Group B	−0.201 (−0.323 ∼ −0.078)	0.002	−0.185 (−0.310 ∼ −0.060)	0.004	−0.158 (−0.286 ∼ −0.029)	0.017
Group C	−0.344 (−0.466 ∼ −0.221)	< 0.001	−0.348 (−0.470 ∼ −0.226)	< 0.001	−0.316 (−0.449 ∼ −0.182)	< 0.001
Group D	−0.525 (−0.648 ∼ −0.403)	< 0.001	−0.512 (−0.637 ∼ −0.387)	< 0.001	−0.459 (−0.595 ∼ −0.324)	< 0.001
Trend	< 0.001	< 0.001	< 0.001	—	—	—

Model I: unadjusted; Model II: adjusted for gender and BMI; Model III: further adjusted for, ALT, ALB, and GFR, One SD, is equal to 12.

BMI, body mass index; ALT, alanine aminotransferase; ALB, albumin; GFR, glomerular filtration rate.

Considering the sample size limitation of the study population and to ensure the robustness of the findings, we constructed models for sensitivity analysis by including different number of variables in the models. For example, in model 1, after adjusting for gender, BMI, ALT, and GFR, the change of propofol requirement in Group B, Group C, and Group D compared to Group A was −0.168 (95%CI = −0.297 ∼ −0.039; *p* = 0.012), −0.340 (95%CI = −0.471 ∼ −0.208; *p* < 0.0001), and -0.490 (95%CI = −0.621 ∼ −0.359; *p* < 0.0001). There was a trend relationship between change in propofol requirement and age (*p* for trend <0.001). There was little change in the other sensitivity analysis results, as shown in the Appendix (Supplementary tables1-3). The results of the sensitivity analysis were consistent with the results of the main analysis.

**FIGURE 2:**
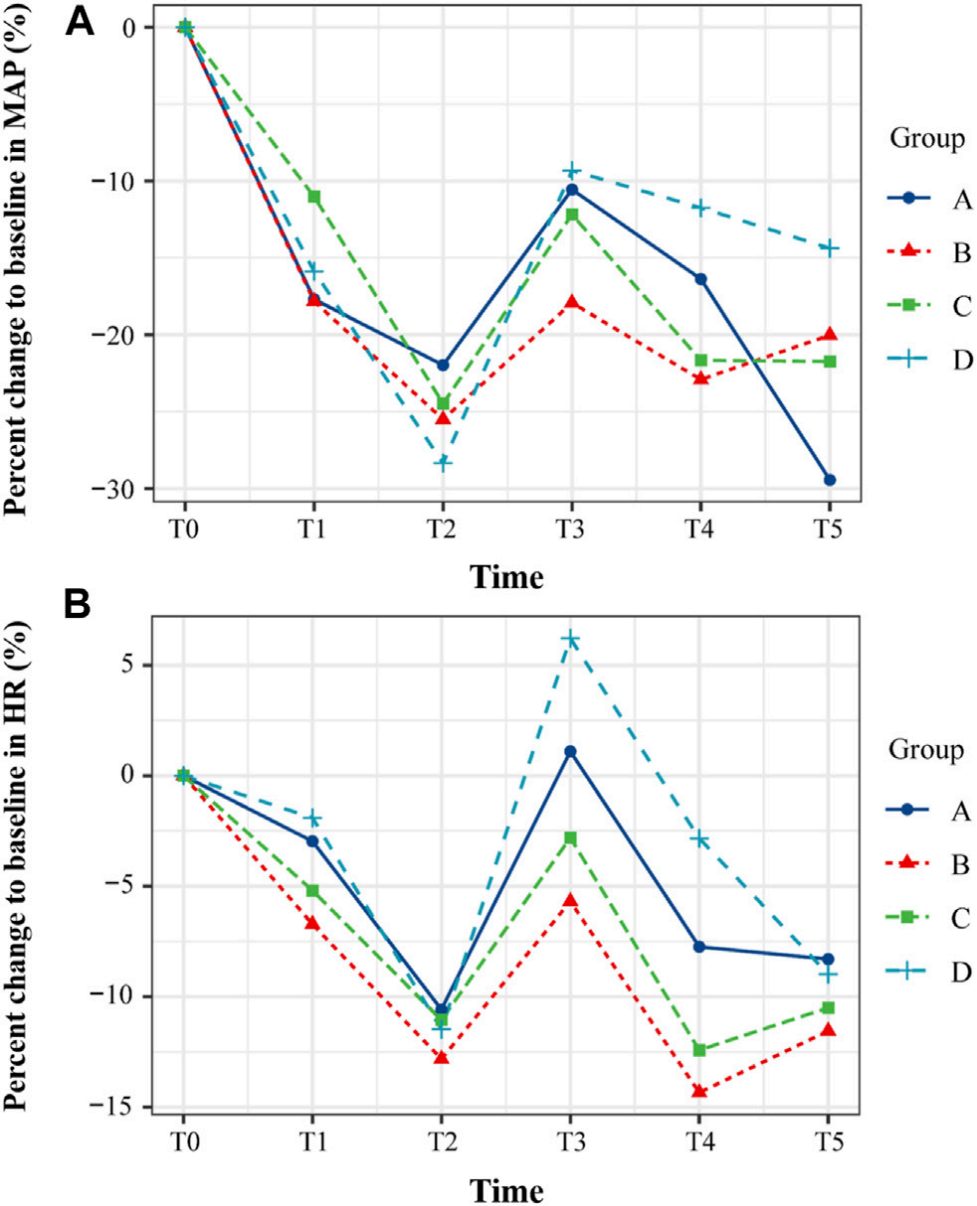
The changes of hemodynamic parameters at five different time points during the induction of anesthesia. **(A)** Mean arterial pressure (MAP). **(B)** Heart rate (HR). The percent changes of MAP and HR relative to the baseline between the four groups were compared. All the values are presented as mean. T0, before propofol administration; T1, LOC; T2, 3 min after the administration of fentanyl and cisatracurium; T3, 1 min after intubation; T4, 5 min after intubation; T5, 10 min after intubation.

After induction of anesthesia, the MAP and HR of patients in each group began to decrease, especially at T2 (3 min after the administration of fentanyl and cisatracurium). After intubation, MAP and HR rebounded in different degrees and tended to be stable in 5–10 min (Figure 2). However, there was no difference in percent changes relative to the baseline between the four groups (MAP: T1, *p* = 0.404; T2, *p* = 0.558; T3, *p* = 0.460; T4, *p* = 0.202; T5, *p* = 0.109; HR: T1, *p* = 0.499; T2, *p* = 0.970; T3, *p* = 0.237; T4, *p* = 0.135; T5, *p* = 0.922).

## Discussion

In the present study, we investigated the effective dose of propofol in surgical patients aged 45 years and over for LOC during the induction of general anesthesia. We found that the propofol requirement for LOC decreased significantly with increasing age. Additionally, we demonstrated that patient age was an independent and significant factor in propofol requirement for LOC, implying that the propofol dose for anesthesia induction should be further reduced in elderly surgical patients, especially those aged 75 years and over.

Increasing aging population paired with age-associated coexisting diseases and longer life spans have resulted in an increasing proportion of geriatric surgery. For these elderly patients, age-related changes in physiology, anatomy and cognitive function have a great impact on both the pharmacodynamics and pharmacokinetics of administered anesthetics (Yang et al., 2011; Alamo et al., 2014; Kok and Reynolds, 2017; Lim and Lee, 2020). Anesthesiologists have to tailor the anesthetic scheme to account the changes associated with aging, comorbidities, and patient medications so as to optimize the perioperative prognosis of these elderly patients. However, guiding evidence focusing on geriatric patients remains poor so far. Clinicians tend to adjust the anesthetic regimen according to their own experience. Additionally, a large retrospective cohort study has found that the median (IQR) propofol dose for anesthesia induction in patients aged over 65 years was 1.8 (1.4–2.2) mg/kg, greater than recommended doses (1–1.5 mg/kg) (Phillips et al., 2015). In our study, the effective dose of propofol for LOC in patients (65–74 years) was 1.15 mk/kg, which is 14.8% lower than that for patients aged 45–64 years. Our findings are in line with previous studies (Koh et al., 2017; You et al., 2019). Moreover, our results show that propofol requirement for LOC in patients aged 75–84 years and ≥85 years are 25.2 and 38.5% lower than that for patients aged ＜ 65 years, respectively (Table 2). Based on age-grouping, we demonstrate that as age increased by decade, the propofol requirement for LOC in elderly reduces dramatically (Figure 1). These findings confirm the guideline for minimal administration of propofol and recommend that the dose of propofol for anesthesia induction should be further reduced for patients aged 75 years and over.

One major purpose of this study was to investigate the impact of age on the propofol requirement for LOC in elderly during anesthesia induction. Therefore, we incorporated factors that might have an impact, such as gender, BMI, albumin, bilirubin, ALT, AST, and GFR into the models (Table 4). After adjustment for these factors, age was still an independent factor. From the perspective of the increased sensitivity of the elderly to anesthetics, our results do not conflict with previous studies (Akhtar, 2018; Kim et al., 2018; Sahinovic et al., 2018; You et al., 2019). However, unlike previous research paradigms, we focused on elderly surgical population which aged over 65 years, and tried to eliminate the interference caused by concomitant medication, comorbidities and renal insufficiency. Remarkably, this geriatric surgical population represents a special group. They typically suffer from cardiopulmonary dysfunction, metabolic diseases, and nervous system dysfunction, etc. (Yang et al., 2011; Lim and Lee, 2020; Pickering et al., 2020). Increasing age represents the change within senescent process, rather than a sort of pathological condition. Correspondingly, this aging process impedes the ability of the body to maintain homeostasis, especially when the body is under stress (Yang et al., 2011; El Beheiry and Mak, 2013). Meanwhile, studies also have shown that advanced age is an independent risk factor for prognosis of various surgical procedures (Keenan and White, 2005; Pommergaard et al., 2016; Tan et al., 2017; Cammarata et al., 2019). Therefore, clinicians should be more aware of these changes caused by aging, so as to provide the most effective perioperative treatment for these elderly surgical patients.

In the present study, Bivariate linear correlation analysis showed that propofol requirement was positively correlated with serum albumin and GFR. It is well known that propofol binds to plasma proteins, mainly serum albumin, because of its lipophilicity. Normally, about 80% of propofol will be bound to serum albumin after intravenous injection (Shityakov et al., 2020), therefore, serum albumin level will significantly affect the pharmacologically active concentration of propofol. GFR is the best test to measure the level of kidney function and determine the stage of kidney disease. GFR declines with age, even in people without kidney disease. Kidneys play an important role in the elimination of propofol. Studies have shown that renal metabolic clearance of propofol accounts for almost one-third of total body clearance and is the major contributor to the extrahepatic elimination of propofol (Hiraoka et al., 2005; Takizawa et al., 2005). However, given that the onset time of propofol is very short (one arm-brain circulation) and the elimination half-life of propofol is quite long (4–6 h), the propofol dose for anesthesia induction is unlikely to be affected by GFR.

In this study, the MAP and HR of the patients decreased gradually with the infusion of propofol, and further decreased with the administration of sufentanil and cisatracurium. Endotracheal intubation reversed the decreasing trend of MAP and HR, and both of them stabilized 10 min later. Elder patients are more prone to hemodynamic instability caused by propofol, due to the increased sensitivity to propofol and the decreased initial distribution volume (Gragasin et al., 2012; You et al., 2019). Concerns on hemodynamic depression of propofol in elderly patients have led anesthesiologists to choose alternative drugs (e.g., etomidate) or to combine with other drugs (e.g., midazolam, dexmedetomidine, etc.) for anesthesia induction. However, a recent study indicates that pretreatment with midazolam and remifentanil led to a significant decrease in MAP, compared with propofol alone (You et al., 2019). There was no case of hypotension or bradycardia in our cohort, which may be due to the slow infusion of propofol on the one hand and the absence of other pretreatment induction drugs on the other. Therefore, in addition to the dosage of propofol, we should also pay attention to the infusion rate of propofol and the choice of combined use of drugs for anesthesia induction in elderly surgical patients.

There are several limitations in our study. First, this was a single-center observational study, with potential selection biases including race, type of surgery, propofol infusion rate. Second, our study only included patients with ASAI∼II, which could eliminate the interference caused by some comorbidities, such as diabetes or hypertension, but it may also limit the universality of our results. Third, each anesthesiologist has his own induction habit such as pretreating with midazolam or dexmedetomidine, which leads to great differences in anesthesia induction.

In addition, some confounding factors that may influence the results might have been overlooked due to unavailable data, including inflammation and nutritional status. Further studies with larger sample sizes, different drug infusion rates, smaller age intervals (e.g., 5 years) and more diverse elderly surgical patients will be needed to more accurately elucidate the relationship between age and propofol induction dose for elderly surgical patients.

In conclusion, this observation study in surgical patients aged 45 years and over demonstrated that age was an independent and significant factor in propofol requirement for LOC during the induction of general anesthesia. Propofol dosage should be tailored in elderly patients, especially those older than 75 years, which may eventually bring benefit to these individuals. However, due to the limitations of our research design, further studies are needed to validate this conclusion and to verify whether this will improve the perioperative prognosis of patients.

## Data Availability

The raw data supporting the conclusion of this article will be made available by the authors, without undue reservation.
