# Supplementary material for: The Impact of Age on Propofol Requirement for Inducing Loss of Consciousness in Elderly Surgical Patients
Source: Front Pharmacol. 2022 Mar 28;13:739552. doi: 10.3389/fphar.2022.739552 (PMC8996377; doi:10.3389/fphar.2022.739552)
Supplement: Supplementary file 1 [file DataSheet1.pdf]

Supplementary table 1: Logistic analysis of age and propofol demand in model I

	β	95% CI	*P*-value	Tolerance	VIF	R^2	Adjusted R^2	*P* for trend*
Age								
Group A	2.612	0.687, 4.537	0.009	UK	UK			
Group B	-0.168	-0.297, -0.039	0.012	0.588	1.702			<0.001
Group C	-0.340	-0.471, -0.208	0.000	0.569	1.757			
Group D	-0.490	-0.621, -0.359	0.000	0.571	1.751			
Male	0.039	-0.048, 0.126	0.370	0.976	1.025	0.544	0.499	
BMI	-0.055	-0.135, 0.025	0.176	0.888	1.126			UK
ALT	-0.001	-0.003, 0.001	0.210	0.866	1.155			
GFR	0.000	-0.001, 0.002	0.561	0.778	1.285			
Per SD increase in age	-0.180	-0.229, -0.131	<0.001	0.825	1.212			
Male	0.040	-0.050, 0.129	0.381	0.976	1.025			
BMI	-0.041	-0.121, 0.039	0.313	0.943	1.060	0.499	0.466	UK
ALT	-0.001	-0.003, 0.001	0.277	0.912	1.097			
GFR	0.000	-0.001, 0.002	0.813	0.794	1.259			

Model I: adjusted for age, gender, BMI, ALT, and GFR.

Abbreviation: BMI, body mass index; ALT, ; GFR, ; CI, confidence interval; VIF, variance inflation factor; UK, UKknown.

* *P* for trend refers to the age group.

Supplementary table 2: Logistic analysis of age and propofol demand in model II

	β	95% CI	*P*-value	Tolerance	VIF	R^2	Adjusted R^2	*P* for trend*
Age, y								
Group A	2.305	0.318, 4.292	0.024	UK	UK			
Group B	-0.159	-0.289, -0.029	0.018	0.576	1.737			<0.001
Group C	-0.317	-0.451, -0.182	0.000	0.538	1.857			
Group D	-0.461	-0.599, -0.323	0.000	0.514	1.945			
Male	0.027	-0.064, 0.118	0.556	0.866	1.129	0.559	0.502	
BMI	-0.054	-0.134, 0.027	0.187	0.878	1.139			
ALT	-0.001	-0.003, 0.001	0.248	0.754	1.327			UK
GFR	0.001	-0.001, 0.002	0.389	0.735	1.361			
ALB	0.006	-0.002, 0.013	0.128	0.829	1.206			
TBIL	0.000	-0.005, 0.006	0.856	0.690	1.450			
Per SD increase in age	-0.168	-0.219, -0.116	<0.001	0.733	1.363			
Male	0.025	-0.069, 0.119	0.596	0.886	1.128			
BMI	-0.041	-0.121, 0.039	0.310	0.935	1.070			
ALT	-0.001	-0.003, 0.001	0.299	0.782	1.279	0.519	0.472	UK
GFR	0.000	-0.001, 0.002	0.560	0.749	1.335			
ALB	0.007	-0.001, 0.014	0.096	0.836	1.196			
TBIL	0.001	-0.005, 0.006	0.799	0.692	1.444			

Model II: adjusted for age, gender, BMI, ALT, GFR, ALB, and TBIL.

Abbreviation: BMI, body mass index; ALT

**P* for trend refers to the age group.

Supplementary table 3: Logistic analysis of age and propofol demand in model III

	β	95% CI	*P*-value	Tolerance	VIF	R^2	Adjusted R^2	*P* for trend*
Age, y								
Group A	2.315	0.317, 4.313	0.024	UK	UK			
Group B	-0.158	-0.289, -0.027	0.019	0.575	1.739			<0.001
Group C	-0.321	-0.457, -0.184	0.000	0.531	1.882			
Group D	-0.467	-0.608, -0.327	0.000	0.498	2.009			
Male	0.029	-0.063, 0.121	0.528	0.878	1.139			
BMI	-0.054	-0.135, 0.026	0.183	0.876	1.141	0.560	0.497	
ALT	-0.002	-0.006, 0.002	0.339	0.143	6.975			
GFR	0.001	-0.001, 0.002	0.374	0.731	1.367			UK
ALB	0.006	-0.002, 0.013	0.136	0.828	1.208			
TBIL	0.000	-0.005, 0.006	0.864	0.698	1.451			
AST	0.001	-0.004, 0.006	0.614	0.144	6.961			
Per SD increase in age	-0.170	-0.224, -0.117	<0.001	0.694	1.441			
Male	0.027	-0.067, 0.122	0.568	0.878	1.139			
BMI	-0.041	-0.121, 0.039	0.313	0.935	1.070			
ALT	-0.002	-0.006, 0.002	0.390	0.145	6.879			
GFR	0.000	-0.001, 0.002	0.552	0.748	1.336	0.520	0.466	UK
ALB	0.006	-0.001, 0.014	0.101	0.835	1.198			
TBIL	0.001	-0.005, 0.006	0.803	0.692	1.444			
AST	0.001	-0.004, 0.006	0.647	0.145	6.903			

Model III: adjusted for gender, BMI, ALT, GFR, ALB, TBIL, and AST.

Abbreviation: BMI, body mass index; ALT

*P for trend refers to the age group.
